# Cardiomyocyte Senescence and Cellular Communications Within Myocardial Microenvironments

**DOI:** 10.3389/fendo.2020.00280

**Published:** 2020-05-21

**Authors:** Xiaoqiang Tang, Pei-Heng Li, Hou-Zao Chen

**Affiliations:** ^1^Key Laboratory of Birth Defects and Related Diseases of Women and Children of MOE, State Key Laboratory of Biotherapy, West China Second University Hospital, Sichuan University, Chengdu, China; ^2^State Key Laboratory of Medical Molecular Biology, Department of Biochemistry and Molecular Biology, Institute of Basic Medical Sciences, Chinese Academy of Medical Sciences and Peking Union Medical College, Beijing, China

**Keywords:** metabolism, cardiomyocytes, senescence, inflammation, microenvironment

## Abstract

Cardiovascular diseases have become the leading cause of human death. Aging is an independent risk factor for cardiovascular diseases. Cardiac aging is associated with maladaptation of cellular metabolism, dysfunction (or senescence) of cardiomyocytes, a decrease in angiogenesis, and an increase in tissue scarring (fibrosis). These events eventually lead to cardiac remodeling and failure. Senescent cardiomyocytes show the hallmarks of DNA damage, endoplasmic reticulum stress, mitochondria dysfunction, contractile dysfunction, hypertrophic growth, and senescence-associated secreting phenotype (SASP). Metabolism within cardiomyocytes is essential not only to fuel the pump function of the heart but also to maintain the functional homeostasis and participate in the senescence of cardiomyocytes. The senescence of cardiomyocyte is also regulated by the non-myocytes (endothelial cells, fibroblasts, and immune cells) in the local microenvironment. On the other hand, the senescent cardiomyocytes alter their phenotypes and subsequently affect the non-myocytes in the local microenvironment and contribute to cardiac aging and pathological remodeling. In this review, we first summarized the hallmarks of the senescence of cardiomyocytes. Then, we discussed the metabolic switch within senescent cardiomyocytes and provided a discussion of the cellular communications between dysfunctional cardiomyocytes and non-myocytes in the local microenvironment. We also addressed the functions of metabolic regulators within non-myocytes in modulating myocardial microenvironment. Finally, we pointed out some interesting and important questions that are needed to be addressed by further studies.

## Introduction

Cardiovascular diseases (CVDs), including cardiomyopathy, heart failure, hypertension, and atherosclerosis, have become the leading cause of death worldwide (1). The continuing increase in CVDs is partially due to the increased number of aging populations. Aging is now considered as a core and independent risk factor for the development of CVDs (2). In aged and pathological myocardial tissues, maladaptation of cellular metabolism, dysfunction (or senescence) of cardiomyocytes, decrease in angiogenesis, and increase in tissue scarring (fibrosis) are observed (3–5). In aged or injured hearts, the senescent cardiomyocytes exhibit the hallmarks of DNA damage, endoplasmic reticulum (ER) stress, mitochondria dysfunction, contractile dysfunction, hypertrophic growth, and senescence-associated secreting phenotype (SASP). The increased senescence of cardiomyocytes centrally contributes to cardiac aging, dysfunction, and failure.

The heart is an organ with high energy demand, and the metabolic pattern of cardiomyocytes is different from the local non-myocytes (3). The mature cardiomyocytes in adult mammalian hearts predominantly use fatty acid oxidation but not glycolysis for energy support (6, 7). During aging and cardiac stress, the alteration in the metabolism of the myocardial tissues is common, which is essentially involved in functional defects of the heart (8, 9). In failing hearts, the cardiomyocytes increase the use of glucose and decrease the use of fatty acid for ATP production, a metabolic pattern dominating in the cardiomyocytes of fetal and neonatal hearts (6). Alteration of metabolism pattern contributes to the senescence of cardiomyocytes and cardiac aging (10).

In addition, the myocardial tissues consist of cardiomyocytes and non-myocytes, including endothelial cells, fibroblasts, and immune cells. Under physiological conditions and developmental progress, the non-myocytes are critically important for the function of cardiomyocytes in the myocardial tissues (11). However, the dysfunction of non-myocytes in the aged and stressed myocardial tissues also facilitates the dysfunction and senescence of cardiomyocytes, enhancing the progress of cardiac diseases (9, 12–16). Cardiomyocytes also modulate the microenvironment by releasing proinflammatory factors, exosomes, and SASPs, which further promote the formation of proinflammation microenvironment and dysfunction of the myocardial tissues (12–14). Metabolic orchestrators also regulate the communications between cardiomyocytes and nonmyocytes within the myocardial microenvironment.

In this review, we will discuss the hallmarks of cardiomyocyte senescence and metabolic modification of senescent cardiomyocytes and the cellular communications within the local microenvironment.

## Senescence of Cardiomyocytes

**Figure 1:**
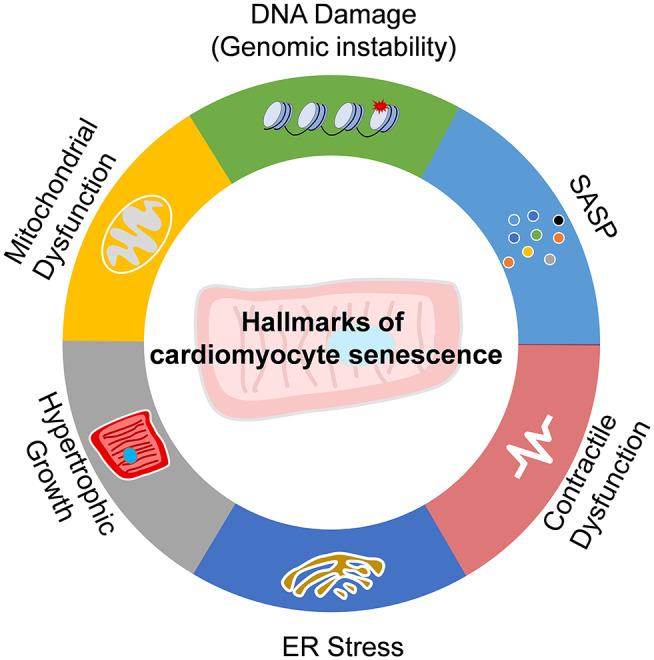
Hallmarks of cardiomyocyte senescence. Senescence of cardiomyocytes exhibits the features of DNA damage and genomic instability, endoplasmic reticulum (ER) stress, mitochondria dysfunction, contractile dysfunction, hypertrophic growth, and senescence-associated secreting phenotype (SASP).

Senescence is a state of cell cycle arrest that promotes tissue remodeling, which contributes to development and injury response. Cellular senescence also contributes to the declines in the regenerative capacity and function of tissues, inducing inflammation, and pathological remodeling in aged organs (15). The cardiomyocytes are terminus-differentiated cells. Cell cycle arrest is not the hallmark of cardiomyocyte senescence. As thus, the senescence of the cardiomyocytes is not easy to give a precise definition as the proliferative or stem cells. Actually, the senescence of cardiomyocytes is generally accomplished with various functional declines, including DNA damage response, ER stress, mitochondria dysfunction, contractile dysfunction, hypertrophic growth, and SASP. Senescent cardiomyocytes also express β-galactosidase (16), which is prominent in senescent cells (15). Here, we summarize these hallmarks in senescent cardiomyocytes (Figure 1).

### DNA Damage

In senescent cardiomyocytes, total cellular and mitochondrial ROS accumulate and induce DNA damage and repair response (17, 18). Telomere shortening is the most common DNA damage feature of senescent cells (19). Evidence in animals and humans documented that post-mitotic cardiomyocyte senescence is mediated by length-independent telomere damage (17, 20). Importantly, telomere-specific DNA damage drives a senescent-like phenotype in cardiomyocytes (17). Therefore, DNA damage is an important driver and hallmark of cardiomyocyte senescence.

### Contractile Dysfunction

In senescent cardiomyocytes, impaired cell shortening and relengthening with increased pacing frequency are intrinsic (21, 22). Contractile dysfunction is also regulated by DNA damage and NAD^+^ depletion (23). Declining cardiac contractile function and changes in metabolism and mitochondrial function contribute to cardiac aging (24).

### ER Stress

In senescent cardiomyocytes with impaired contractility, ER stress is accumulated. The unfolded protein response is a prominent feature of ER stress (25). ER stress also contributes to apoptosis and hypertrophic growth of cardiomyocytes (26, 27). Attenuation of ER stress prevents cardiomyocyte senescence and improves cardiomyocyte contractility cardiac function (28, 29).

### Mitochondrial Dysfunction

In senescent cardiomyocytes, the fission–fusion progress of mitochondria is imbalanced and the function is declined. Mitochondrial dysfunction is the key feature of cardiomyocyte senescence (17, 30). P53 inhibits cyclin-dependent kinases and plays an important role in cell-cycle arrest in senescent cells. P53 also inhibits Parkin-mediated mitophagy and promotes mitochondrial dysfunction to facilitate cellular senescence (31). Improvement of mitochondria function *via* targeting Drp1/Parkin/PINK1 signaling can repress the senescence of cardiomyocytes (30, 32).

### Senescence-Associated Secreting Phenotype (SASP)

Senescent cells secrete different factors, including pro-inflammatory cytokines and chemokines, growth modulators, angiogenetic factors, and matrix metalloproteinases (MMPs) (33). Senescent cardiomyocytes increased expression of SASP factors including CCN family member 1 (CCN1), interleukins (IL1α, IL1β, and IL6), tumor necrosis factor-alpha (TNFα), and monocyte chemoattractant protein-1 (MCP1), endothelin 3 (Edn3), tumor growth factor-beta (TGFβ), and growth and differentiation factor 15 (GDF15) (17, 34). These SASP factors play important roles in regulating non-myocytes within the local microenvironment and contribute to cardiac remodeling and dysfunction.

### Hypertrophic Growth

In myocardial tissues of aged rodents and humans, one of the key features of dysfunctional cardiomyocytes is the pathologically hypertrophic growth. Senescence is commonly associated with hypertrophic growth in cardiomyocytes (34). Cardiomyocyte hypertrophy is inhibited when inhibitory components of cell cycle regulators are activated (35). In senescent cardiomyocytes, enhanced expression of hypertrophic genes and enlarged cell size are observed (17).

Cardiomyocyte senescence is typical in cardiac aging and diseases. For instance, chemical drugs such as doxorubicin can induce premature senescence in cardiomyocytes, with positive staining of senescence-associated β-galactosidase, CDK-I expression, decreased cardiac troponin I phosphorylation, and decreased telomerase activity. This senescence phenotype was associated with the acetylation of p53, the key protein involved in stress-induced premature senescence in proliferating cells (35). The senescence of cardiomyocytes leads to cardiac hypertrophy, arrhythmia, and other types of cardiomyopathy. Importantly, the senescence of cardiomyocytes is critically involved in cardiac aging and late stage of cardiac remodeling and heart failure (18).

The senescence of cardiomyocytes is regulated by the intracellular signaling pathways such as the metabolic sensors/regulators and the extracellular microenvironment, such as the paracrine effects of the non-myocytes (endothelial cells and fibroblasts, as well as immune cells). Here, we will discuss the modulation of metabolism and local microenvironment on cardiomyocyte senescence.

## The Regulation of Metabolism Dysfunction on Cardiomyocyte Senescence

The hearts in mammals beat unstoppably, which needs a large amount of energy. The metabolism pattern in the cardiomyocytes is much different from other cells, and the pattern changes with development, physiological, and pathological responses (36). In addition, the metabolism pattern of mammalian hearts and cardiomyocytes also alter with aging. The dysfunction of metabolism in cardiomyocytes is a pivotal contributor to the senescence of cardiomyocytes and the functional decline of the heart (10).

The cardiomyocytes use fatty acid and glucose for their energy support predominantly. Fatty acyl-coenzyme A (CoA) and pyruvate, which are metabolites of fatty acid oxidation and glucose oxidation pathways, are the major resources for the production of energy (ATP) in mitochondria of cardiomyocytes. The entry of long-chain acyl-CoA into mitochondrion is regulated by carnitine–palmitoyl transferase-1 (CPT1) reaction, and the oxidation of pyruvate is regulated by pyruvate dehydrogenase (PDH) (6). CPT1 and PDH are rate-limiting enzymes of these two pathways in mitochondria. For instance, the level of CPT1 is significantly decreased in the heart tissue of aging rats (37). The decline of CPT1 during aging might lead to cardiac complications in pathologic conditions (38). The deficiency of CPT1 aggravates cardiomyocyte senescence and pressure-overload-induced cardiac hypertrophy due to lipotoxicity (39). In addition, peroxisome proliferator-activated receptor α (PPARα) and PGC-1α are also essential regulators of fatty acid metabolism. The expression of PPARα and PGC-1α level declines with age (40). In an experimental murine model of aging, decreased PPARα mRNA and protein levels increased ceramide levels, which was associated with cardiac hypertrophy in senescence-accelerated prone mice (41). Insulin signaling was necessary for glucose metabolism in cardiomyocytes. Activation of insulin signaling *via* insulin growth factor receptor (IGFR) in cardiomyocytes induces SASP and promotes cardiomyocyte senescence (16). Activation of P53 facilitates glycolysis to promote cardiomyocyte senescence and inhibition of P53 prevents cardiomyocyte senescence and diabetic cardiomyopathy (42).

Ketone bodies are the minor substrates for oxidative metabolism in cardiomyocytes. Increased ketone bodies, including acetoacetate, β-hydroxybutyrate, and acetone, have been observed in the heart of aging and heart failure patients (43, 44). Ketone bodies are likely beneficial for the function of hearts. In age-associated cardiac hypertrophy and failure, the energy source shifts to ketone bodies for oxidative ATP production, which reduces oxidative and inflammatory damages in cardiomyocytes (44). β-Hydroxybutyrate is the primary ketone body produced by the body during ketosis. β-Hydroxybutyrate improves cardiomyocyte excitation–contraction coupling, protects the cells against hypoxic stress and represses cardiomyocyte senescence (45). Cardiomyocyte-specific deficiency of ketone body metabolism by knocking out of succinyl-CoA:3-oxoacid CoA transferase (SCOT) promotes mitochondria stress and cell senescence to accelerate pathological remodeling (46).

The hexosamine biosynthetic pathway also regulates the senescence of cardiomyocytes. Fructose 6-phosphate, the glycolytic intermediate, can diverge into the hexosamine biosynthetic pathway by the enzyme glutamine fructose 6-phosphate amidotransferase (GFAT) (47). Uridine diphosphate-N-acetylglucosamine (UDP-GlcNAc) is produced and used as the substrate of O-linked-GlcNAc transferase (OGT), which catalyzes the O-GlcNAcylation (O-GlcNAc) of proteins (48). Protein O-GlcNAcylation plays an essential role as a protective response in cardiomyocyte senescence *via* reducing calcium overload, mitochondrial permeability transition pore opening, ER stress, modification of inflammatory and heat shock responses (49). Of note, the cardiac protective effects of increasing O-GlcNAc levels have been emphasized in many pathologic conditions, including ischemic injury (50, 51).

In addition to the substrate metabolic pathways, the core metabolic regulators such as AMP-activated protein kinase (AMPK), NAD+-dependent Sirtuins, FOXOs, and mammalian target of rapamycin (mTOR) also regulate the senescence of cardiomyocytes. For instance, AMPK regulates both glucose and fatty acid metabolism *via* modulating Acetyl-CoA carboxylase (ACC) and glucose transporter GLUT4. AMPK activation was reduced in aged myocardial tissues, and activation of AMPK improves mitochondrial dynamics, reduces ER stress, and improves the function of the cardiomyocytes to repress cardiomyocyte senescence (52–54). The effects of AMPK are partially due to the results of reduced protein O-GlcNAcylation (54). The Sirtuins are NAD^+^-dependent regulators of cellular metabolism and senescence. SIRT1, SIRT2, SIRT3, SIRT6, and SIRT7 are reported to regulate cardiac aging (13, 55–59). SIRT2 targets the Liver kinase B1 (LKB1)-AMPK signaling to regulate energetic metabolic and hypertrophic growth of cardiomyocytes in aged mice (53). In addition, SIRT3 and SIRT4 cooperate to modulate ROS metabolism in the mitochondria to regulate hypertrophic growth of senescent cardiomyocytes (60).

Collectively, cellular metabolism is essential for the homeostasis of cardiomyocytes and participates in cardiomyocyte senescence during aging and the development of various diseases.

## Local Microenvironment and Cardiomyocyte Senescence

**Figure 2:**
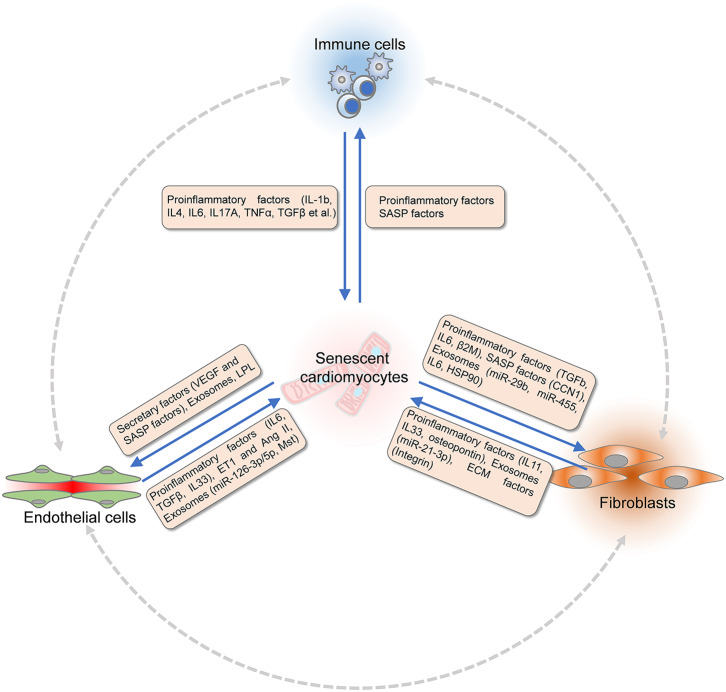
Communications between cardiomyocytes and non-myocytes within the microenvironment. Signals released by non-myocytes contribute to cardiomyocytes senescence. The dysfunctional endothelial cells (ECs) secrete pro-inflammatory factors (TGF-β, IL-6, and IL-33), ET-1, and Ang II to modulate cardiomyocyte senescence. Extracellular ventricles (EVs) or exosomes produced by ECs that consist of Mst1 and microRNAs also regulate the function of cardiomyocytes. Fibroblasts modulate cardiomyocyte senescence by paracrine signaling and remodeling of extracellular matrix (ECM). Fibroblasts express matrix metalloproteinases (MMPs), integrins, and fibronectin to interact with ECM, which are critical for paracrine signaling. Proinflammatory factors including IL-11, IL-33, and EVs composed of miR-21-3p, osteopontin, and EGFR are secreted by fibroblasts to modulate the senescence of cardiomyocytes. Different signals released by various immune cells modulate cardiomyocytes senescence directly. Reciprocally, senescence of dysfunctional cardiomyocytes undergoes SASP. Proinflammatory factors and chemokines are produced to recruit immune cells. Senescence-associated secretory factors, VEGF, LPL, and EVs are released by cardiomyocytes to induce senescence and dysfunction of ECs under age-associated pathological conditions. Similarly, the function of fibroblasts is regulated by paracrine factors from dysfunctional cardiomyocytes under stress conditions. TGF-β, transforming growth factor-beta; IL-6, interleukin 6; IL-33, interleukin 33; Ang II, angiotensin II; EGFR, epithelial cell growth factor receptor; SASP, senescence-associated secretory phenotype; VEGF, vascular endothelial growth factor; LPL, lipoprotein lipase.

Aging and age-associated pathologic conditions lead to remodeling of the local microenvironment. Different components interact with each other and lead to a dysfunctional heart in the pathological conditions. Reciprocally, signals released by non-myocytes cells also affect cardiomyocytes and contribute to cardiomyocyte senescence (Figure 2).

### The Interaction Between Endothelial Cells and Cardiomyocytes

During aging and disease progress, the metabolism of endothelial cells also changes. Although the mitochondria content in endothelial cells is low, the functions of metabolic regulators and metabolites are critical for the function of endothelial cells, which was reviewed and discussed in our previous review paper and others (36, 61). Endothelial cells not only serve as the basic layer of the vasculature but also secrete paracrine factors to modulate the cellular microenvironment. The dysfunction of endothelial cells facilitates the dysfunction and senescence of cardiomyocytes. The interaction between endothelial cells and cardiomyocytes is complex. Here, we mainly discussed the paracrine functions of endothelial cells in cardiomyocyte senescence.

Endothelial cells secrete endothelial-specific paracrine factors and inflammatory factors contributing to cardiomyocyte senescence as well as cardiac dysfunction. During embryonic and postnatal development, endothelial cells secrete platelet-derived growth factor (PDGF)–B, angiotensin II (Ang II), prostacyclin (PGI2), prostaglandin E2 (PGE2), parathyroid hormone-related peptide (PTHRP), nitric oxide (NO), endothelin 1 (ET-1), and neuregulin-1 (NRG1) to promote the proliferation and maturation of cardiomyocytes (62). In the condition of metabolic dysfunction and cardiac remodeling, endothelial cells secrete Ang II, NRG1, ET-1, apelin, and proinflammatory factors (e.g., TGFβ and IL6), and eicosanoids to modulate the senescence of cardiomyocytes (62). For instance, high expression and release of Ang II and ET-1 by endothelial cells can stimulate the mitochondrial dysfunction, ER stress, contractional dysfunction, and hypertrophic growth of cardiomyocytes *via* binding their receptors on cardiomyocytes. In the cardiovascular system, ET-1 is the most potent vasoconstrictor that has remarkably long-lasting actions. ET-1 is an important contributor to vasoconstriction, vascular and cardiac hypertrophy, as well as tissue inflammation, and subsequently participates in the development and progression of cardiomyocyte senescence and CVDs (63, 64). The upregulation of Ang II and ET-1 promoted age-associated cardiac hypertrophy and fibrosis (53, 65). By contrast, depletion of endothelin receptor A (ETAR) in cardiomyocytes rescues aging-associated or high-fat-diet-induced cardiomyocyte hypertrophy and contractile dysfunction by regulating autophagy response (66, 67). Interleukin 33 (IL-33) is a pro-inflammatory factor. Endothelial cell secretion of IL-33 is crucial for the translation of myocardial pressure overload into inflammatory responses *via* binding to membrane-bound ST2 (ST2L) on cardiomyocytes (68).

Cardiac endothelial cells also produce exosomes or extracellular vehicles to affect cardiomyocyte functions. Exosome Mst1 derived from endothelial cells inhibits autophagy and promotes apoptosis in cardiomyocytes *via* inhibiting the binding between Daxx and GLUT4 and repressing glucose metabolism under the diabetic condition (69). The exosomes from endothelial cells also contain microRNAs (e.g., miRNA-126-3p and−5p) that regulate the function of cardiomyocytes (70).

The effects of endothelial cells on cardiomyocyte senescence are also regulated by metabolism. Liver kinase B1 (LKB1) is a central regulator of cell polarity and energy homeostasis by activating the central metabolic regulator AMPK (71). Endothelial cell-specific LKB1 deletion causes endothelial dysfunction. These endothelial cells cause hypertension and induce cardiomyocyte hypertrophy (72). LKB1-AMPK signaling may also contribute to cardiomyocyte senescence *via* the paracrine pathway. AMPK in endothelial cells regulates the release of NO and inflammatory factors to contribute to tissue remodeling (72, 73). Therefore, endothelial metabolism is critical for cardiac remodeling.

In summary, endothelial cells secrete angiocrine and proinflammatory factors to modulate the maturation, hemostasis, and senescence of cardiomyocytes, which are also regulated by the metabolic pattern of endothelial cells. In addition, the endothelial cells also transdifferentiate to mesenchymal cells in the endocardial cushion through the endothelial-to-mesenchymal transition (EndoMT), a process that is critical for cardiomyocyte functions and contributes cardiac development and diseases as discussed elsewhere (74–76).

### Fibroblast Contributes to Cardiomyocyte Senescence

Fibroblasts are key components of the healthy cardiac tissues and are emerging as a pivotal regulator of cardiac function *via* intramyocardial fibroblast–cardiomyocyte communications (77). Fibroblasts not only contribute to cardiac development and homeostasis but also are critically involved in cardiac remodeling and diseases.

Cardiac fibroblasts secrete paracrine factors such as Ang II, cardiotrophin 1, fibroblast growth factor (FGF), IL-6, insulin-like growth factor 1 (IGF1), TGFβ, and TNFα to mediate fibroblast–cardiomyocyte communications (12). Similar to endothelial cells, fibroblasts also secrete IL-33 to reduce cardiomyocyte senescence induced by hypertrophic and hypoxic injuries (77). Recently, IL-11 was identified as a paracrine factor of fibroblasts in the heart tissues. IL-11 secreted by the fibroblasts contributes to cardiomyocyte dysfunction and cardiac hypertrophy, as well as a functional decline of the heart (78). The profibrotic roles of IL11 were also observed in the kidney, liver, and lung (78–80). In addition, the plasma membrane calcium ATPase 4 signaling in cardiac fibroblasts mediates cardiomyocyte hypertrophy *via* upregulating the expression and release of frizzled-related protein 2 (sFRP2) (81).

In addition, fibroblast exosomes also regulate cardiomyocytes. microRNAs are enriched in exosomes derived from cardiac fibroblasts. Fibroblast exosome–derived miR-21_3p serves as a potent paracrine-acting microRNA that induces cardiomyocyte hypertrophy in rodents by targeting Sorbin and SH3 domain-containing protein 2 (SORBS2) and PDZ and LIM domain 5 (PDLIM5) (82). Angiotensin II treatment of fibroblasts enhanced the release of exosomes with osteopontin and epidermal growth factor receptor (EGFR), which activates the renin–angiotensin system and enhances cardiomyocyte hypertrophy (83). Exosomes from human cardiac fibroblasts also modulate calcium cycling of cardiomyocytes by abbreviating cytoplasmic calcium transient duration (84). Further studies are needed to identify which components of the exosome derived from fibroblasts contributed to cardiomyocyte dysfunction and senescence.

Cardiac fibroblasts modulate cardiomyocyte senescence *via* paracrine functions and remodeling of extracellular matrix (ECM) (11). Fibroblasts express integrins and matrix metalloproteinases (MMPs) to modulate the local extracellular matrix. For instance, fibroblast-expressed integrins provide critical adhesive and signaling functions by directly interacting with the ECM and the actin cytoskeleton, which are critical for the paracrine signaling, ECM homeostasis, and for the intercellular interactions that regulate cardiomyocyte senescence and pathological adaptations (12, 85). Fibronectin polymerization is necessary for collagen matrix deposition. Fibronectin is a central contributor to the increased abundance of cardiac myofibroblasts during cardiac injury and remodeling. Fibronectin polymerization inhibitor (pUR4) or genetic deletion of fibronectin in fibroblasts repressed the cardiomyocyte hypertrophy and heart failure (86). Cardiac calcification is common with age and injury, which leads to heart blocks. Cardiac fibroblasts adopt an osteoblast cell-like fate and regulate cardiomyocyte calcification directly (87).

Metabolic modulators regulate the function of fibroblasts in cardiac aging and remodeling. For instance, adiponectin activates the APPL1-AMPK signaling and induces cell migration, MMP activation, and collagen remodeling in cardiac fibroblasts (88). AMPK activation increases the content of fibroblasts in the infarcted area (89). FOXO3A mediates Peroxiredoxin III expression, which plays a critical role in the resistance to oxidative stress in cardiac fibroblasts. In addition, FoxO1 contributes to TGFβ-dependent cardiac myofibroblast differentiation (90). As thus, metabolic regulations within cardiac fibroblasts may also participate fundamentally in regulating the dysfunction and senescence of cardiomyocytes.

### Immune Cells Contribute to Cardiomyocyte Senescence

Immune cells play a crucial role in tissue homeostasis and pathogenesis. Immune cells in the cardiac tissues can regulate inflammatory response and modulate cardiomyocyte senescence directly. These immune cells include macrophages, T cells, and mast cells.

A large number of studies have identified the roles of macrophages in the physiological and pathological progress of cardiac tissues. Macrophages participate in cardiac development, regeneration, and pathological remodeling (91, 92). Cardiac macrophages promote the electrical conduction of cardiomyocytes through the distal atrioventricular node, where conducting cells closely intersperse with elongated Connexin 43-positive macrophages. Macrophage-specific depletion of Connexin 43 induces cardiomyocyte senescence with dysfunctional electrical activity (93). Cardiac macrophages with NLRP3 inflammasome activation secreted IL-1β, which promotes cardiomyocyte senescence by causing prolongation of the action potential duration, inducing a decrease in potassium current and an increase in calcium sparks in cardiomyocytes of diabetic mice (94). A similar role of macrophages was also observed in atrial fibrillation (95). Iron regulator hepcidin impairs macrophage-dependent cardiac repair after injury. *Hepcidin* deficiency increased the content of chemokine (C-C motif) receptor 2 (CCR2)^+^ inflammatory macrophages. These macrophages fostered signal transducer and activator of transcription factor-3 (STAT3) phosphorylation to release IL-4 and IL-13, and to favor cardiomyocyte renewal (96). Macrophage-mediated inflammation also contributes to cardiomyocyte hypertrophy (11). Tissue-resistant macrophages also contribute to the recruitment and activation of other immune cells (97). Therefore, macrophages are critically involved in cardiomyocyte renewal and senescence. However, it remains to be documented which subtypes of macrophages contribute to self-renewal and which subtypes of macrophages contribute to cardiomyocyte senescence, and the specific paracrine factors are also needed to be investigated.

T cells play a central role in adaptive immunity, but their functional roles in cardiac pathophysiology are still poorly understood. δT lymphocytes predominantly produce IL-17A. During myocardial ischemia/reperfusion, IL-17A promotes cardiomyocyte apoptosis (98). T cell costimulation blockade inhibits cardiomyocyte apoptosis and blunts pressure overload-induced hypertrophic growth of cardiomyocytes and heart failure (99). Adoptively transferred CD4^+^CD25^+^ regulatory T (Treg) cells ameliorate Ang II-induced cardiac damage and fibrosis in the hypertensive heart (100). Recently, a study reported the paracrine functions of Treg in cardiac damage. Upon myocardial infraction, Treg cells secrete Cystatin F, TNF superfamily member 11 (TNFSF11), IL-33, fibrinogen-like protein 2 (FGL-2), matrilin-2, and IGF-2 to promote cardiomyocyte proliferation and represses cardiomyocyte senescence (101).

In addition to macrophages and T cells, mast cells are involved in inflammation and tissue remodeling. The effects of mast cells on cardiomyocyte dysfunction are also significant. After coronary microembolization, mast cell contributes to cardiomyocyte apoptosis (102). Deficiency of mast cells leads to depressed cardiomyocyte contractility and reduced postischemic cardiac function caused by myofilament Ca^2+^ desensitization (103). Mast cell-derived IL-6 and TNF-α promote cardiomyocyte death and diabetic cardiomyopathy (104). In addition, mast cells release chymase to promote the production of TGFβ to aggravate cardiomyocyte hypertrophy and senescence (105). Therefore, mast cell is an essential regulator of cardiomyocyte function and senescence via paracrine pathways.

Metabolic factors are critical for the activation of immune cells. For instance, metabolic reprogramming contributes to macrophage polarization. Classically activated or type I macrophages (M1) are known to obtain energy through glycolysis. By contrast, alternatively activated or type II macrophages (M2) use oxidative metabolism to fuel their longer-term functions in tissue repair and wound healing (106). Both AMPK and SIRT1 have involved macrophage metabolism and inflammatory activation. AMPK promotes macrophage fatty acid oxidative metabolism to mitigate inflammatory activation of macrophages and subsequently participates in aging-related CVDs (107). Our previous findings show that SIRT1 regulates the polarization of macrophages and participate in cardiovascular aging (108, 109). Glucose and fatty acid metabolism are also critically involved in the differentiation, survival, and activation of T cells and mast cell, where the metabolic orchestrators AMPK, SIRT1, and FOXP3 also play regulatory roles (110–113). To this point, metabolism regulators and metabolites in immune cells, such as macrophages, also contribute to cardiomyocyte dysfunction and senescence, but further studies are needed to verify this hypothesis.

## The Effects of Senescent Cardiomyocytes on Nonmyocytes

The cells within the myocardial microenvironment communicate with each other to modular cardiac homeostasis and aging. Non-myocytes can regulate the physiological functions and senescence of cardiomyocytes. By contrast, cardiomyocytes also affect the functions of non-myocytes partially *via* a paracrine manner. Here, we mainly focus on the effects of cardiomyocytes on endothelial cells and fibroblasts/myofibroblasts.

### The Effects of Senescent Cardiomyocytes on Endothelial Cells

The effects of cardiomyocytes on endothelial cells are significant. Cardiomyocytes can secrete angiogenic factors to facilitate the survival, proliferation, and angiogenesis of endothelial cells (Figure 2). For instance, cardiomyocytes produce and release vascular endothelial cell growth factor A (VEGFA) and angiopoietin-1 to promote endothelial survival and angiogenesis (114). VEGF secreted by cardiomyocytes binds VEGF receptor 2 (VEGFR2) on endothelial cells to promote angiogenesis (115). Additional extensively studied cardiokines inducing cardiac angiogenesis include the other VEGF family members VEGF-B, VEGF-C, and placental growth factor (PlGF), as well as FGFs, hepatocyte growth factor (HGF), and angiopoietin-1 (116).

Dysfunctional or senescent cardiomyocytes undergo SASP, which upregulated senescence-related secretory factors, including CCN family member 1 (CCN1), IL-1α, TNF-α, TGF-β, and monocyte chemoattractant protein (34). The SASP factors released by cardiomyocytes can induce dysfunction and senescence of endothelial cells. Of note, cardiomyocytes also regulate EndMT. Other than myofibroblasts, cardiomyocytes are another critical resource of TGF-β, the key regulator of EndMT (117). TGF-β released by cardiomyocytes can trigger the EndMT progress, which is essential for cardiac development and represents a key feature of dysfunctional myocardial tissues (117, 118).

Lipoprotein lipase (LPL) is secreted by dysfunctional cardiomyocytes in diabetes (119, 120). Glycosylphosphatidylinositol-anchored high-density lipoprotein binding protein (GPIHBP1) at the basolateral side of the endothelial cells captured LPL and transferred it across to the epical side. LPL–GPIHBP1 complex on endothelial cells hydrolyzed lipoprotein-TG and released fatty acid (121, 122). As a result, more fatty acids could be delivered to diabetic cardiomyocytes. VEGFA also has paracrine effects on endothelial cells. VEGFA activates Notch signaling, resulting in enhanced GPIHB1 expression and increased LPL translocation across the endothelial cells (123).

Exosomes or extracellular vesicles also contribute to the intercellular cardiomyocyte–endothelial cell communications. Exosome secreted by cardiomyocytes enriched in HSP20 was found to promote proliferation, migration, and tube formation of human umbilical vein endothelial cells (HUVECs) by activating VEGFR2 (124). MicroRNAs are also enriched in exosomes from cardiomyocytes. In rats with type 2 diabetes, dysfunctional cardiomyocytes can produce miR-320-enriched exosomes to modulate endothelial cells. These exosomes reduce the proliferation, migration, and tube formation of endothelial cells *via* downregulation of IGF-1, HSP20, and ETS2, impairing angiogenesis in diabetic hearts (125).

Collectively, cardiomyocytes can release angiogenic factors, LPL, exosomes, as well as SASP factors to modulate the function of endothelial cells.

### The Effects of Dysfunctional Cardiomyocytes on Fibroblasts

Cardiac fibrosis was considered as the result of a reparative process activated in response to cardiomyocyte injury (Figure 2). Aging and metabolic perturbations, such as diabetes and obesity, might cause interstitial and perivascular fibrosis in the absence of infarction (126–128). Activated fibroblasts or myofibroblasts are the main effector cells in cardiac fibrosis. Dysfunctional cardiomyocytes could modulate fibroblasts.

Under stress conditions, cardiomyocytes can produce and secrete a wide range of paracrine factors to modulate the function of fibroblasts. These paracrine factors include interleukins, TGF-β, CCN1, β-2 microglobulin (β2M), FGF, placental growth factor (PGF), as well as danger-associated molecular patterns. In cardiac fibroblasts, TGF-β is centrally involved in many aspects of fibrosis, including myofibroblast activation and ECM remodeling. The expression of TGF-β in cardiomyocytes increases in both dilated and hypertrophic cardiomyopathies (12). Apical resection induced cardiomyocytes to secrete the matricellular protein CCN1. CCN1 results in fibroblast senescence, which promotes neonatal heart regeneration by enhancing cardiomyocyte proliferation and reducing cardiac fibrosis (129). IL-6 produced by cardiomyocytes can activate fibroblasts *via* the STAT-3 signaling (130, 131). Cardiomyocytes also produce connective tissue growth factor (CTGF), which can promote myofibroblast activation. Mechanical stretch induced the rapid secretion of β2M, a non-glycosylated protein related to inflammatory diseases, mainly from cardiomyocytes. The paracrine β2M from cardiomyocytes activates cardiac fibroblasts *via* epidermal growth factor receptor (EGFR) (132). PGF plays critical roles in the heart as a paracrine regulator of cardiac adaptation to stress stimulation. Upon stress, cardiomyocytes secrete PGF, which activates fibroblasts to induce the paracrine function of fibroblasts and further hypertrophic growth of cardiomyocytes (133). After myocardial infarction, necrotic cardiomyocytes release danger-associated molecular patterns (DAMPs) to induce a proinflammatory phenotype in fibroblasts, inducing the secretion of cytokines and chemokines and stimulating leukocyte infiltration (134).

Exosomes from impaired cardiomyocytes also interfere with fibroblasts and might result in excessive and uncontrolled fibrosis. Hypertrophic myocytes released exosomes enriched in inflammatory cytokines and non-coding RNAs. IL-6 in exosomes derived from cardiomyocytes activates STAT-3 signaling in fibroblasts, which leads to collagen production and deposition during cardiac hypertrophy (135). Lifestyle intervention might change the deleterious effects of exosomes. Under the condition of diabetes, exercise increased miR-29b and miR-455 in exosomes, reducing cardiac fibrosis *via* downregulating MMP9 in the diabetic heart (136). MiR-378 is preferentially expressed in cardiomyocytes. Cardiac mechanical stress makes cardiomyocytes secrete more exosomes enriched with miR-378, which are transported into the cardiac fibroblasts. In cardiac fibroblasts, miR-378 regulates the p38 MAPK-Smad2/3 signaling pathway by targeting MMK6 and then inhibits fibrosis (132). Other microRNAs in cardiomyocyte exosomes include miR92a (137). In addition, long non-coding RNA-enriched vesicles secreted by hypoxic cardiomyocytes also promote the activation of fibroblasts and drive cardiac fibrosis (95).

The work in our lab demonstrated that metabolic dysfunction in cardiomyocytes also induced the activation of fibroblasts. Sirtuins are NAD+-dependent histone deacetylases related to longevity and metabolism. It connected enzyme activity, metabolism, and aging. We observed that SIRT2 overexpression in cardiomyocytes activated the metabolic LKB1–AMPK pathway and reduced aging-associated hypertrophy or senescence of cardiomyocytes, which leads to the repression of fibrosis (53). In addition, SIRT3 and SIRT4 cooperate to regulate ROS metabolism in the mitochondria to maintain the homeostasis of myocardial tissues by repressing cardiomyocyte hypertrophy and, subsequently, activation of fibroblasts (60).

## Conclusion and Perspectives

The heart is an organ with high energy demand. Different from the non-myocytes, mitochondria content in cardiomyocytes is up to 70%. Metabolic homeostasis is very important for the development and physiological function of cardiomyocytes. Compensatory metabolic dynamics in cardiomyocytes contribute to the functional balance of the heart. During aging and stress conditions, the metabolic pattern changes in cardiomyocytes, which is critically involved in the regulation of cardiomyocyte dysfunction and senescence. The non-myocytes (endothelial cells, fibroblasts, and immune cells) in the local microenvironment also contribute to the (dys)function/senescence of cardiomyocytes. In turn, the senescent cardiomyocytes modulate the microenvironment to contribute to functional compensatory response or decompensatory remodeling and cardiac dysfunction.

However, our understanding of the myocardial microenvironment and cardiomyocyte senescence is still poor. Further studies are still needed to elucidate the following questions (1). Although we have summarized some features/hallmarks of senescent cardiomyocytes, the clear definition of cardiomyocyte senescence is needed and the core mechanisms underlying cardiomyocyte senescence are not clear. Specifically, it remains to elucidate how remodeling of chromatin structure and DNA released by cardiomyocytes contribute to the senescence of cardiomyocytes (2). Although cell senescence plays essential roles in wound healing, limiting atherosclerotic plaque size, and preventing infections, the effects of cell senescence can be detrimental or beneficial. The exact roles of senescent cells that contribute to aging and age-related diseases can be named “senescaging,” as we described previously. Senescaging elucidates how senescent cells lead to organism aging and eventually to age-related diseases (2, 138). Therefore, further studies are still needed to explore the physiological and pathological functions of senescent cardiomyocyte during cardiac development, regeneration, and pathological remodeling, and to elucidate how senescaging contributes to cardiac aging and disease. Specifically, more studies are needed to answer whether cardiomyocyte senescence critically contributes to cardiac aging and the related heart failure with preserved ejection fraction (HFpEF) (3). Microenvironmental non-myocytes function as central regulators of cardiomyocyte senescence, and metabolism switch is important for the homeostasis and senescence of cardiomyocytes. As thus, an interesting point is whether these non-myocytes affect the metabolic pattern of cardiomyocyte undergoing senescence. Also, studies are needed to explore how metabolism alternations in non-myocytes contribute to cardiomyocyte senescence and cardiac aging. Martin and colleagues reported an extensive transcriptome study of the process of heart aging in a rat model, focusing primarily on inflammatory and immune signals. They suggested that the process of heart aging was not identical in males and females, which may be primarily due to the variant conditions of hormone and metabolism between males and females. This is an aspect that is likely to be of substantial clinical interest in the future (139) (4). Many studies have been carried out to study the effects of non-myocytes on cardiomyocyte senescence. Some studies also explored the paracrine effects of cardiomyocytes on non-myocytes. However, our knowledge about the effects of senescent cardiomyocytes on microenvironmental non-myocytes is few and further efforts are needed (5). The fifth but not last interesting question is whether cardiomyocyte senescence and the myocardial microenvironment could serve as targets for anti-aging drugs such as the popular senolytics. Senolytics was recently reported to repress senescence and inhibit cardiac disease such as myocardial infarction (140) and repress age-related vasomotor dysfunction and atherosclerosis (141). Further studies are still needed to elucidate how senolytics target cardiomyocyte senescence and local microenvironment, and that whether other anti-aging drugs could repress the senescaging of myocardial microenvironment.

## Author Contributions

XT and H-ZC conceptualized the review. XT and P-HL wrote the manuscript with the help from H-ZC.

## Conflict of Interest

The authors declare that the research was conducted in the absence of any commercial or financial relationships that could be construed as a potential conflict of interest.

## References

[1] Miranda JJ Barrientos-Gutierrez T Corvalan C Hyder AA Lazo-Porras M Oni T. Understanding the rise of cardiometabolic diseases in low- and middle-income countries. Nat Med. (2019) 25:1667–79. 10.1038/s41591-019-0644-7 31700182

[2] Ding YN Tang X Chen HZ Liu DP. Epigenetic regulation of vascular aging and age-related vascular diseases. Adv Exp Med Biol. (2018) 1086:55–75. 10.1007/978-981-13-1117-8_4 30232752

[3] Picca A Mankowski RT Burman JL Donisi L Kim JS Marzetti E. Mitochondrial quality control mechanisms as molecular targets in cardiac ageing. Nat Rev Cardiol. (2018) 15:543–54. 10.1038/s41569-018-0059-z 30042431 PMC6283278

[4] Roh J Rhee J Chaudhari V Rosenzweig A. The role of exercise in cardiac aging: from physiology to molecular mechanisms. Circ Res. (2016) 118:279–95. 10.1161/CIRCRESAHA.115.305250 26838314 PMC4914047

[5] Gude NA Broughton KM Firouzi F Sussman MA. Cardiac ageing: extrinsic and intrinsic factors in cellular renewal and senescence. Nat Rev Cardiol. (2018) 15:523–42. 10.1038/s41569-018-0061-5 30054574

[6] Kolwicz SC Jr Purohit S Tian R. Cardiac metabolism and its interactions with contraction, growth, and survival of cardiomyocytes. Circ Res. (2013) 113:603–16. 10.1161/CIRCRESAHA.113.302095 23948585 PMC3845521

[7] Chen XF Chen X Tang X. Short-chain fatty acid, acylation and cardiovascular diseases. Clin Sci. (2020) 134:657–76. 10.1042/CS20200128 32219347

[8] Heusch G Libby P Gersh B Yellon D Böhm M Lopaschuk G. Cardiovascular remodelling in coronary artery disease and heart failure. Lancet. (2014) 383:1933–43. 10.1016/S0140-6736(14)60107-0 24831770 PMC4330973

[9] Patel KV Pandey A Lemos JA. Conceptual framework for addressing residual atherosclerotic cardiovascular disease risk in the era of precision medicine. Circulation. (2018) 137:2551–3. 10.1161/CIRCULATIONAHA.118.035289 29643058

[10] Li H Hastings MH Rhee J Trager LE Roh JD Rosenzweig A. Targeting age-related pathways in heart failure. Circ Res. (2020) 126:533–51. 10.1161/CIRCRESAHA.119.315889 32078451 PMC7041880

[11] Kamo T Akazawa H Komuro I. Cardiac nonmyocytes in the hub of cardiac hypertrophy. Circ Res. (2015) 117:89–98. 10.1161/CIRCRESAHA.117.305349 26089366

[12] Saucerman JJ Tan PM Buchholz KS McCulloch AD Omens JH. Mechanical regulation of gene expression in cardiac myocytes and fibroblasts. Nat Rev Cardiol. (2019) 16:361–78. 10.1038/s41569-019-0155-8 30683889 PMC6525041

[13] Tang X Chen XF Chen HZ Liu DP. Mitochondrial Sirtuins in cardiometabolic diseases. Clin Sci. (2017) 131:2063–78. 10.1042/CS20160685 28739840

[14] Costantino S Paneni F Cosentino F. Ageing, metabolism and cardiovascular disease. J Physiol. (2016) 594:2061–73. 10.1113/JP270538 26391109 PMC4933114

[15] Hernandez-Segura A Nehme J Demaria M. Hallmarks of cellular senescence. Trends Cell Biol. (2018) 28:436–53. 10.1016/j.tcb.2018.02.001 29477613

[16] Ock S Lee WS Ahn J Kim HM Kang H Kim HS. Deletion of IGF-1 receptors in cardiomyocytes attenuates cardiac aging in male mice. Endocrinology. (2016) 157:336–45. 10.1210/en.2015-1709 26469138 PMC4701888

[17] Anderson R Lagnado A Maggiorani D Walaszczyk A Dookun E Chapman J. Length-independent telomere damage drives post-mitotic cardiomyocyte senescence. EMBO J. (2019) 38:e100492. 10.15252/embj.2018100492 30737259 PMC6396144

[18] Mitry MA Laurent D Keith BL Sira E Eisenberg CA Eisenberg LM. Accelerated cardiomyocyte senescence contributes to late-onset doxorubicin-induced cardiotoxicity. Am J Physiol Cell Physiol. (2020) 318:C380–91. 10.1152/ajpcell.00073.2019 31913702 PMC7052608

[19] Shay JW Wright WE. Telomeres and telomerase: three decades of progress, Nature reviews. Genetics. (2019) 20:299–309. 10.1038/s41576-019-0099-1 30760854

[20] Ball AJ Levine F. Telomere-independent cellular senescence in human fetal cardiomyocytes. Aging Cell. (2005) 4:21–30. 10.1111/j.1474-9728.2004.00137.x 15659210

[21] Lim CC Apstein CS Colucci WS Liao R. Impaired cell shortening relengthening with increased pacing frequency are intrinsic to the senescent mouse cardiomyocyte. J Mol Cell Cardiol. (2000) 32:2075–82. 10.1006/jmcc.2000.1239 11040110

[22] Yang X Doser TA Fang CX Nunn JM Janardhanan R Zhu M. Metallothionein prolongs survival and antagonizes senescence-associated cardiomyocyte diastolic dysfunction: role of oxidative stress. FASEB J. (2006) 20:1024–6. 10.1096/fj.05-5288fje 16585059

[23] Zhang D Hu X Li J Liu J Baks-te Bulte L Wiersma M. DNA damage-induced PARP1 activation confers cardiomyocyte dysfunction through NAD+ depletion in experimental atrial fibrillation. Nat Commun. (2019) 10:1307. 10.1038/s41467-019-09014-2 30898999 PMC6428932

[24] Barton GP de Lange WJ Ralphe JC Aiken J Diffee G. Linking metabolic and contractile dysfunction in aged cardiac myocytes. Physiol Rep. (2017) 5:e13485. 10.14814/phy2.13485 29084842 PMC5661240

[25] Groenendyk J Agellon LB Michalak M. Coping with endoplasmic reticulum stress in the cardiovascular system. Annu Rev Physiol. (2013) 75:49–67. 10.1146/annurev-physiol-030212-183707 23020580

[26] Xie F Wu D Huang SF Cao JG Li HN He L. The endoplasmic reticulum stress-autophagy pathway is involved in apelin-13-induced cardiomyocyte hypertrophy *in vitro*. Acta Pharmacol Sin. (2017) 38:1589–600. 10.1038/aps.2017.97 28748915 PMC5719161

[27] Zeng Z Huang N Zhang Y Wang Y Su Y Zhang H. CTCF inhibits endoplasmic reticulum stress and apoptosis in cardiomyocytes by upregulating RYR2 via inhibiting S100A1. Life Sci. (2020) 242:117158. 10.1016/j.lfs.2019.117158 31837328

[28] Bozi LH Takano AP Campos JC Rolim N Dourado PM Voltarelli VA. Endoplasmic reticulum stress impairs cardiomyocyte contractility through JNK-dependent upregulation of BNIP3. Int J Cardiol. (2018) 272:194–201. 10.1016/j.ijcard.2018.08.070 30173922

[29] Wiersma M Meijering RA Qi XY Zhang D Liu T Hoogstra-Berends F. Endoplasmic reticulum stress is associated with autophagy and cardiomyocyte remodeling in experimental and human atrial fibrillation. J Am Heart Assoc. (2017) 6:e006458. 10.1161/JAHA.117.006458 29066441 PMC5721854

[30] Nishimura A Shimauchi T Tanaka T Shimoda K Toyama T Kitajima N. Hypoxia-induced interaction of filamin with Drp1 causes mitochondrial hyperfission–associated myocardial senescence. Sci Signal. (2018) 11:eaat5185. 10.1126/scisignal.aat5185 30425165

[31] Hoshino A Mita Y Okawa Y Ariyoshi M Iwai-Kanai E Ueyama T. Cytosolic p53 inhibits Parkin-mediated mitophagy and promotes mitochondrial dysfunction in the mouse heart. Nat Commun. (2013) 4:2308. 10.1038/ncomms3308 23917356

[32] Ren X Chen L Xie J Zhang Z Dong G Liang J. Resveratrol ameliorates mitochondrial elongation via Drp1/Parkin/PINK1 signaling in senescent-like cardiomyocytes. Oxid Med Cell Longev. (2017) 2017:4175353. 10.1155/2017/4175353 29201272 PMC5671746

[33] Coppe JP Desprez PY Krtolica A Campisi J. The senescence-associated secretory phenotype: the dark side of tumor suppression. Annu Rev Pathol. (2010) 5:99–118. 10.1146/annurev-pathol-121808-102144 20078217 PMC4166495

[34] Cui S Xue L Yang F Dai S Han Z Liu K. Postinfarction hearts are protected by premature senescent cardiomyocytes via GATA4‐dependent CCN1 secretion. J Am Heart Assoc. (2018) 7:e009111. 10.1161/JAHA.118.009111 30371213 PMC6222958

[35] Maejima Y Adachi S Ito H Hirao K Isobe M. Induction of premature senescence in cardiomyocytes by doxorubicin as a novel mechanism of myocardial damage. Aging Cell. (2008) 7:125–36. 10.1111/j.1474-9726.2007.00358.x 18031568

[36] Tang X Luo YX Chen HZ Liu DP. Mitochondria, endothelial cell function, vascular diseases. Front Physiol. (2014) 5:175. 10.3389/fphys.2014.00175 24834056 PMC4018556

[37] Zhang X Liu C Liu C Wang Y Zhang W Xing Y. Trimetazidine and lcarnitine prevent heart aging and cardiac metabolic impairment in rats via regulating cardiac metabolic substrates. Exp Gerontol. (2019) 119:120–7. 10.1016/j.exger.2018.12.019 30639303

[38] Bogazzi F Raggi F Ultimieri F Russo D D'Alessio A Manariti A. Regulation of cardiac fatty acids metabolism in transgenic mice overexpressing bovine GH. J Endocrinol. (2009) 201:419–27. 10.1677/JOE-08-0194 19342398

[39] Long Q Liu J Wang P Zhou Y Ding Y Prasain J. Carnitine Palmitoyltransferase-1b (CPT1b) deficiency aggravates pressure-overload-induced cardiac hypertrophy due to lipotoxicity. Circulation. (2012) 126:1705–16. 10.1161/CIRCULATIONAHA.111.075978 22932257 PMC3484985

[40] Dillon LM Rebelo AP Moraes CT. The role of PGC-1 coactivators in aging skeletal muscle and heart. IUBMB Life. (2012) 64:231–41. 10.1002/iub.608 22279035 PMC4080206

[41] Rodriguez-Calvo R Serrano L Barroso E Coll T Palomer X Camins A. Peroxisome proliferator-activated receptor alpha down-regulation is associated with enhanced ceramide levels in age-associated cardiac hypertrophy. J Gerontol. (2007) 62:1326–36. 10.1093/gerona/62.12.1326 18166682

[42] Gu J Wang S Guo H Tan Y Liang Y Feng A. Inhibition of p53 prevents diabetic cardiomyopathy by preventing early-stage apoptosis and cell senescence, reduced glycolysis, impaired angiogenesis. Cell Death Dis. (2018) 9:82. 10.1038/s41419-017-0093-5 29362483 PMC5833384

[43] Aubert G Martin OJ Horton JL Lai L Vega RB Leone TC. The failing heart relies on ketone bodies as a fuel. Circulation. (2016) 133:698–705. 10.1161/CIRCULATIONAHA.115.017355 26819376 PMC4766035

[44] Bedi KC Jr Snyder NW Brandimarto J Aziz M Mesaros C Worth AJ. Evidence for intramyocardial disruption of lipid metabolism and increased myocardial ketone utilization in advanced human heart failure. Circulation. (2016) 133:706–16. 10.1161/CIRCULATIONAHA.115.017545 26819374 PMC4779339

[45] Klos M Morgenstern S Hicks K Suresh S Devaney EJ. The effects of the ketone body β-hydroxybutyrate on isolated rat ventricular myocyte excitation-contraction coupling. Arch Biochem Biophys. (2019) 662:143–50. 10.1016/j.abb.2018.11.027 30543786

[46] Schugar RC Moll AR Andre d'Avignon D Weinheimer CJ Kovacs A Crawford PA. Cardiomyocyte-specific deficiency of ketone body metabolism promotes accelerated pathological remodeling. Mol Metab. (2014) 3:754–69. 10.1016/j.molmet.2014.07.010 25353003 PMC4209361

[47] Hebert LF Jr Daniels MC Zhou J Crook ED Turner RL Simmons ST. Overexpression of glutamine:fructose-6-phosphate amidotransferase in transgenic mice leads to insulin resistance. J Clin Invest. (1996) 98:930–6. 10.1172/JCI118876 8770864 PMC507507

[48] Wells L Vosseller K Hart GW. Glycosylation of nucleocytoplasmic proteins: signal transduction and O-GlcNAc. Science. (2001) 291:2376–8. 10.1126/science.1058714 11269319

[49] Jensen RV Andreadou I Hausenloy DJ Botker HE. The role of O-GlcNAcylation for protection against ischemia-reperfusion injury. Int J Mol Sci. (2019) 20:404. 10.3390/ijms20020404 30669312 PMC6359045

[50] Champattanachai V Marchase RB Chatham JC. Glucosamine protects neonatal cardiomyocytes from ischemia-reperfusion injury via increased protein O-GlcNAc and increased mitochondrial Bcl-2. Am J Physiol Cell Physiol. (2008) 294:C1509–20. 10.1152/ajpcell.00456.2007 18367586 PMC2800950

[51] Jones SP Zachara NE Ngoh GA Hill BG Teshima Y Bhatnagar A. Cardioprotection by N-acetylglucosamine linkage to cellular proteins. Circulation. (2008) 117:1172–82. 10.1161/CIRCULATIONAHA.107.730515 18285568

[52] Turdi S Fan X Li J Zhao J Huff AF Du M. AMP-activated protein kinase deficiency exacerbates aging-induced myocardial contractile dysfunction. Aging Cell. (2010) 9:592–606. 10.1111/j.1474-9726.2010.00586.x 20477759 PMC2910211

[53] Tang X Chen XF Wang NY Wang XM Liang ST Zheng W. SIRT2 acts as a cardioprotective deacetylase in pathological cardiac hypertrophy. Circulation. (2017) 136:2051–67. 10.1161/CIRCULATIONAHA.117.028728 28947430 PMC5698109

[54] Gélinas R Mailleux F Dontaine J Bultot L Demeulder B Ginion A. AMPK activation counteracts cardiac hypertrophy by reducing O-GlcNAcylation. Nat Commun. (2018) 9:1–17. 10.1038/s41467-017-02795-4 29371602 PMC5785516

[55] Kane AE Sinclair DA. Sirtuins and NAD+ in the development and treatment of metabolic and cardiovascular diseases. Circ Res. (2018) 123:868–85. 10.1161/CIRCRESAHA.118.312498 30355082 PMC6206880

[56] Vakhrusheva O Smolka C Gajawada P Kostin S Boettger T Kubin T. Sirt7 increases stress resistance of cardiomyocytes and prevents apoptosis and inflammatory cardiomyopathy in mice. Circ Res. (2008) 102:703–10. 10.1161/CIRCRESAHA.107.164558 18239138

[57] Tang X Ma H Han L Zheng W Lu YB Chen XF. SIRT1 deacetylates the cardiac transcription factor Nkx2.5 and inhibits its transcriptional activity. Sci Rep. (2016) 6:36576. 10.1038/srep36576 27819261 PMC5098195

[58] Hsu YJ Hsu SC Hsu CP Chen YH Chang YL Sadoshima J. Sirtuin 1 protects the aging heart from contractile dysfunction mediated through the inhibition of endoplasmic reticulum stress-mediated apoptosis in cardiac-specific Sirtuin 1 knockout mouse model. Int J Cardiol. (2017) 228:543–52. 10.1016/j.ijcard.2016.11.247 27875732

[59] Sundaresan NR Vasudevan P Zhong L Kim G Samant S Parekh V. The sirtuin SIRT6 blocks IGF-Akt signaling and development of cardiac hypertrophy by targeting c-Jun. Nat Med. (2012) 18:1643–50. 10.1038/nm.2961 23086477 PMC4401084

[60] Luo YX Tang X An XZ Xie XM Chen XF Zhao X. Sirt4 accelerates Ang II-induced pathological cardiac hypertrophy by inhibiting manganese superoxide dismutase activity. Eur Heart J. (2017) 38:1389–98. 10.1093/eurheartj/ehw138 27099261

[61] Eelen G de Zeeuw P Simons M Carmeliet P. Endothelial cell metabolism in normal and diseased vasculature. Circ Res. (2015) 116:1231–44. 10.1161/CIRCRESAHA.116.302855 25814684 PMC4380230

[62] Colliva A Braga L Giacca M Zacchigna S. Endothelial cell–cardiomyocyte crosstalk in heart development and disease. J Physiol. (2019). 10.1113/JP276758 30816576 PMC7496632

[63] Dhaun N Webb DJ. Endothelins in cardiovascular biology and therapeutics. Nat Rev Cardiol. (2019) 16:491–502. 10.1038/s41569-019-0176-3 30867577

[64] Cowling RT. The aging heart, endothelin-1 the senescent cardiac fibroblast. J Mol Cell Cardiol. (2015) 81:12–4. 10.1016/j.yjmcc.2015.01.018 25647277

[65] Wang X Guo Z Ding Z Khaidakov M Lin J Xu Z. Endothelin-1 upregulation mediates aging-related cardiac fibrosis. J Mol Cell Cardiol. (2015) 80:101–9. 10.1016/j.yjmcc.2015.01.001 25584774

[66] Ceylan-Isik AF Dong M Zhang Y Dong F Turdi S Nair S. Cardiomyocyte-specific deletion of endothelin receptor A rescues aging-associated cardiac hypertrophy and contractile dysfunction: role of autophagy. Basic Res Cardiol. (2013) 108:335. 10.1007/s00395-013-0335-3 23381122 PMC3590116

[67] Ceylan AF Wang S Kandadi MR Chen J Hua Y Pei Z. Cardiomyocyte-specific knockout of endothelin receptor a attenuates obesity cardiomyopathy. Biochim Biophys Acta. (2018) 1864:3339–52. 10.1016/j.bbadis.2018.07.020 30031229

[68] Chen WY Hong J Gannon J Kakkar R Lee RT. Myocardial pressure overload induces systemic inflammation through endothelial cell IL-33. Proc Natl Acad Sci USA. (2015) 112:7249–54. 10.1073/pnas.1424236112 25941360 PMC4466705

[69] Hu J Wang S Xiong Z Cheng Z Yang Z Lin J. Exosomal Mst1 transfer from cardiac microvascular endothelial cells to cardiomyocytes deteriorates diabetic cardiomyopathy. Biochim Biophys Acta. (2018) 1864:3639–49. 10.1016/j.bbadis.2018.08.026 30251683

[70] Akbar N Digby JE Cahill TJ Tavare AN Corbin AL Saluja S. Endothelium-derived extracellular vesicles promote splenic monocyte mobilization in myocardial infarction. JCI Insight. (2017) 2:e93344. 10.1172/jci.insight.93344 28878126 PMC5621885

[71] Herzig S Shaw RJ. AMPK: guardian of metabolism and mitochondrial homeostasis. Nat Rev Mol Cell Biol. (2017) 19:121–35. 10.1038/nrm.2017.95 28974774 PMC5780224

[72] Zhang W Wang Q Wu Y Moriasi C Liu Z Dai X. Endothelial Cell–specific liver kinase B1 deletion causes endothelial dysfunction and hypertension in mice *in vivo*. Circulation. (2014) 129:1428–39. 10.1161/CIRCULATIONAHA.113.004146 24637557 PMC3972325

[73] Omura J Satoh K Kikuchi N Satoh T Kurosawa R Nogi M. Protective roles of endothelial AMP-activated protein kinase against hypoxia-induced pulmonary hypertension in mice. Circ Res. (2016) 119:197–209. 10.1161/CIRCRESAHA.115.308178 27217398

[74] Li Y Lui KO Zhou B. Reassessing endothelial-to-mesenchymal transition in cardiovascular diseases. Nat Rev Cardiol. (2018) 15:445–56. 10.1038/s41569-018-0023-y 29748594

[75] Jackson AO Zhang J Jiang Z Yin K. Endothelial-to-mesenchymal transition: a novel therapeutic target for cardiovascular diseases. Trends Cardiovasc Med. (2017) 27:383–93. 10.1016/j.tcm.2017.03.003 28438397

[76] Kovacic JC Dimmeler S Harvey RP Finkel T Aikawa E Krenning G. Endothelial to mesenchymal transition in cardiovascular disease. JACC. (2019) 73:190–209. 10.1016/j.jacc.2018.09.089 30654892 PMC6865825

[77] Kakkar R Lee RT. Intramyocardial fibroblast myocyte communication. Circ Res. (2010) 106:47–57. 10.1161/CIRCRESAHA.109.207456 20056945 PMC2805465

[78] Schafer S Viswanathan S Widjaja AA Lim WW Moreno-Moral A DeLaughter DM. IL-11 is a crucial determinant of cardiovascular fibrosis. Nature. (2017) 552:110–5. 10.1038/nature24676 29160304 PMC5807082

[79] Widjaja AA Singh BK Adami E Viswanathan S Dong J D'Agostino GA. Inhibiting interleukin 11 signaling reduces hepatocyte death and liver fibrosis, inflammation, and steatosis in mouse models of nonalcoholic steatohepatitis. Gastroenterology. (2019) 157:777–92.e14. 10.1053/j.gastro.2019.05.002 31078624

[80] Ng B Dong J D'Agostino G Viswanathan S Widjaja AA Lim WW. Interleukin-11 is a therapeutic target in idiopathic pulmonary fibrosis. Sci Transl Med. (2019) 11:eaaw1237. 10.1126/scitranslmed.aaw1237 31554736

[81] Mohamed MA Abou-Leisa R Stafford N Maqsood A Zi M Prehar S. The plasma membrane calcium ATPase 4 signalling in cardiac fibroblasts mediates cardiomyocyte hypertrophy. Nat Commun. (2016) 7:11074. 10.1038/ncomms11074 27020607 PMC4820544

[82] Bang C Batkai S Dangwal S Gupta SK Foinquinos A Holzmann A. Cardiac fibroblast–derived microRNA passenger strand-enriched exosomes mediate cardiomyocyte hypertrophy. J Clin Invest. (2014) 124:2136–46. 10.1172/JCI70577 24743145 PMC4001534

[83] Lyu L Wang H Li B Qin Q Qi L Nagarkatti M. A critical role of cardiac fibroblast-derived exosomes in activating renin angiotensin system in cardiomyocytes. J Mol Cell Cardiol. (2015) 89:268–79. 10.1016/j.yjmcc.2015.10.022 26497614 PMC4988239

[84] Wang BX Couch L MacLeod KT Harding SE Terracciano CM Extracellular vesicles secreted from human fibroblasts modulate human induced pluripotent stem cell- cardiomyocyte calcium cycling. Circulation. (2017) 136:A19928.

[85] Civitarese RA Kapus A McCulloch CA Connelly KA. Role of integrins in mediating cardiac fibroblast–cardiomyocyte cross talk: a dynamic relationship in cardiac biology and pathophysiology. Basic Res Cardiol. (2016) 112:6. 10.1007/s00395-016-0598-6 28000001

[86] Valiente-Alandi I Potter SJ Salvador AM Schafer AE Schips T Carrillo-Salinas F. Inhibiting fibronectin attenuates fibrosis and improves cardiac function in a model of heart failure. Circulation. (2018) 138:1236–52. 10.1161/CIRCULATIONAHA.118.034609 29653926 PMC6186194

[87] Pillai CL Li S Romay M Lam L Lu Y Huang J. Cardiac fibroblasts adopt osteogenic fates and can be targeted to attenuate pathological heart calcification. Cell Stem Cell. (2017) 20:218–32.e5. 10.1016/j.stem.2016.10.005 27867037 PMC5291784

[88] Dadson K Chasiotis H Wannaiampikul S Tungtrongchitr R Xu A Sweeney G. Adiponectin mediated APPL1-AMPK signaling induces cell migration, MMP activation, and collagen remodeling in cardiac fibroblasts. Cell Biochem. (2014) 115:785–93. 10.1002/jcb.24722 24255018

[89] Cieslik KA Taffet GE Crawford JR Trial J Mejia Osuna P Entman ML. AICAR-dependent AMPK activation improves scar formation in the aged heart in a murine model of reperfused myocardial infarction. Mol Cell Cardiol. (2013) 63:26–36. 10.1016/j.yjmcc.2013.07.005 23871790 PMC3820161

[90] Vivar R Humeres C Muñoz C Boza P Bolivar S Tapia F. FoxO1 mediates TGF-beta1-dependent cardiac myofibroblast differentiation, Biochim. Biophys Acta. (2016) 1863:128–38. 10.1016/j.bbamcr.2015.10.019 26518453

[91] Aurora AB Porrello ER Tan W Mahmoud AI Hill JA Bassel-Duby R. Macrophages are required for neonatal heart regeneration. Clin Invest. (2014) 124:1382–92. 10.1172/JCI72181 24569380 PMC3938260

[92] Frangogiannis NG. Emerging roles for macrophages in cardiac injury: cytoprotection, repair, and regeneration. J Clin Invest. (2015) 125:2927–30. 10.1172/JCI83191 26214519 PMC4563767

[93] Hulsmans M Clauss S Xiao L Aguirre AD King KR Hanley A. Macrophages facilitate electrical conduction in the heart. Cell. (2017) 169:510–22.e20. 10.1016/j.cell.2017.03.050 28431249 PMC5474950

[94] Monnerat G Alarcon ML Vasconcellos LR Hochman-Mendez C Brasil G Bassani RA. Macrophage-dependent IL-1beta production induces cardiac arrhythmias in diabetic mice. Nat Commun. (2016) 7:13344. 10.1038/ncomms13344 27882934 PMC5123037

[95] Sun Z Zhou D Xie X Wang S Wang Z Zhao W. Cross-talk between macrophages and atrial myocytes in atrial fibrillation. Basic Res Cardiol. (2016) 111:63. 10.1007/s00395-016-0584-z 27660282 PMC5033992

[96] Zlatanova I Pinto C Bonnin P Mathieu JRR Bakker W Vilar J. Iron regulator hepcidin impairs macrophage-dependent cardiac repair after injury. Circulation. (2019) 139:1530–47. 10.1161/CIRCULATIONAHA.118.034545 30586758

[97] Bajpai G Bredemeyer A Li W Zaitsev K Koenig AL Lokshina I. Tissue resident CCR2- and CCR2+ cardiac macrophages differentially orchestrate monocyte recruitment and fate specification following myocardial injury. Circ Res. (2019) 124:263–78. 10.1161/CIRCRESAHA.118.314028 30582448 PMC6626616

[98] Liao YH Xia N Zhou SF Tang TT Yan XX Lv BJ. Interleukin-17A contributes to myocardial ischemia/reperfusion injury by regulating cardiomyocyte apoptosis and neutrophil infiltration. Am Coll Cardiol. (2012) 59:420–9. 10.1016/j.jacc.2011.10.863 22261166 PMC3262985

[99] Kallikourdis M Martini E Carullo P Sardi C Roselli G Greco CM. T cell costimulation blockade blunts pressure overload-induced heart failure. Nat Commun. (2017) 8:14680. 10.1038/ncomms14680 28262700 PMC5343521

[100] Kvakan H Kleinewietfeld M Qadri F Park JK Fischer R Schwarz I. Regulatory T cells ameliorate angiotensin II–induced cardiac damage. Circulation. (2009) 119:2904–12. 10.1161/CIRCULATIONAHA.108.832782 19470887

[101] Zacchigna S Martinelli V Moimas S Colliva A Anzini M Nordio A. Paracrine effect of regulatory T cells promotes cardiomyocyte proliferation during pregnancy and after myocardial infarction. Nat Commun. (2018) 9:2432. 10.1038/s41467-018-04908-z 29946151 PMC6018668

[102] Zhang QY Ge JB Chen JZ Zhu JH Zhang LH Lau CP. Mast cell contributes to cardiomyocyte apoptosis after coronary microembolization. J Histochem Cytochem. (2006) 54:515–23. 10.1369/jhc.5A6804.2005 16344327

[103] Ngkelo A Richart A Kirk JA Bonnin P Vilar J Lemitre M. Mast cells regulate myofilament calcium sensitization and heart function after myocardial infarction. J Exp Med. (2016) 213:1353–74. 10.1084/jem.20160081 27353089 PMC4925026

[104] He A Fang W Zhao K Wang Y Li J Yang C. Mast cell-deficiency protects mice from streptozotocin-induced diabetic cardiomyopathy. Transl Res. (2019) 208:1–14. 10.1016/j.trsl.2019.01.005 30738862 PMC6527494

[105] Li J Jubair S Janicki JS. Estrogen inhibits mast cell chymase release to prevent pressure overload-induced adverse cardiac remodeling. Hypertension. (2015) 65:328–34. 10.1161/HYPERTENSIONAHA.114.04238 25403608 PMC4289018

[106] Galván-Peña S O'Neill LAJ. Metabolic reprograming in macrophage polarization. Front Immunol. (2014) 5:420. 10.3389/fimmu.2014.00420 25228902 PMC4151090

[107] Steinberg GR Schertzer JD. AMPK promotes macrophage fatty acid oxidative metabolism to mitigate inflammation: implications for diabetes and cardiovascular disease. Immunol Cell Biol. (2014) 92:340–5. 10.1038/icb.2014.11 24638063

[108] Zhang R Chen HZ Liu JJ Jia YY Zhang ZQ Yang RF. SIRT1 suppresses activator protein-1 transcriptional activity cyclooxygenase-2 expression in macrophages. J Biol Chem. (2010) 285:7097–110. 10.1074/jbc.M109.038604 20042607 PMC2844159

[109] Zhang Z Xu J Liu Y Wang T Pei J Cheng L. Mouse macrophage specific knockout of SIRT1 influences macrophage polarization and promotes angiotensin II-induced abdominal aortic aneurysm formation. J Genet Genom. (2018) 45:25–32. 10.1016/j.jgg.2018.01.002 29396144

[110] Pearce EL Walsh MC Cejas PJ Harms GM Shen H Wang LS. Enhancing CD8 T-cell memory by modulating fatty acid metabolism. Nature. (2009) 460:103–7. 10.1038/nature08097 19494812 PMC2803086

[111] Angelin A Gil-de-Gómez L Dahiya S Jiao J Guo L Levine MH. Foxp3 reprograms T cell metabolism to function in low-glucose, high-lactate environments. Cell Metab. (2017) 25:1282–93.e7. 10.1016/j.cmet.2016.12.018 28416194 PMC5462872

[112] Li X Lee YJ Jin F Park YN Deng Y Kang Y. Sirt1 negatively regulates FcεRI-mediated mast cell activation through AMPK-and PTP1B-dependent processes. Sci Rep. (2017) 7:6444. 10.1038/s41598-017-06835-3 28744004 PMC5527079

[113] Li X Park SJ Jin F Deng Y Yang JH Chang JH. Tanshinone IIA suppresses FcεRI-mediated mast cell signaling and anaphylaxis by activation of the Sirt1/LKB1/AMPK pathway. Biochem Pharmacol. (2018) 152:362–72. 10.1016/j.bcp.2018.04.015 29674003

[114] Hsieh PCH Davis ME Lisowski LK Lee RT. Endothelial-cardiomyocyte interactions in cardiac development and repair. Annu Rev Physiol. (2006) 68:51–66. 10.1146/annurev.physiol.68.040104.124629 16460266 PMC2754585

[115] Ueda K Toko H Komuro I. Endothelial Cell–derived angiocrines elicit physiological cardiomyocyte hypertrophy. Circulation. (2019) 139:2585–7. 10.1161/CIRCULATIONAHA.119.040632 31136219

[116] Talman V Kivelä R. Cardiomyocyte—endothelial cell interactions in cardiac remodeling and regeneration. Front Cardiovasc Med. (2018) 5:101. 10.3389/fcvm.2018.00101 30175102 PMC6108380

[117] Zeisberg EM Tarnavski O Zeisberg M Dorfman AL McMullen JR Gustafsson E. Endothelial-to-mesenchymal transition contributes to cardiac fibrosis. Nat Med. (2007) 13:952–61. 10.1038/nm1613 17660828

[118] Song S Liu L Yu Y Zhang R Li Y Cao W. Inhibition of BRD4 attenuates transverse aortic constriction- and TGF-β-induced endothelial-mesenchymal transition and cardiac fibrosis. J Mol Cell Cardiol. (2019) 127:83–96. 10.1016/j.yjmcc.2018.12.002 30529267

[119] Wang Y Zhang D Chiu AP Wan A Neumaier K Vlodavsky I. Endothelial heparanase regulates heart metabolism by stimulating lipoprotein lipase secretion from cardiomyocytes. Arterioscler Thromb Vasc Biol. (2013) 33:894–902. 10.1161/ATVBAHA.113.301309 23471235

[120] Zhang D Wan A Chiu AP Wang Y Wang F Neumaier K. Hyperglycemia-induced secretion of endothelial heparanase stimulates a vascular endothelial growth factor autocrine network in cardiomyocytes that promotes recruitment of lipoprotein lipase. Arterioscler Thromb Vasc Biol. (2013) 33:2830–8. 10.1161/ATVBAHA.113.302222 24115032

[121] Dallinga-Thie GM Franssen R Mooij HL Visser ME Hassing HC Peelman F. The metabolism of triglyceride-rich lipoproteins revisited: new players, new insight. Atherosclerosis. (2010) 211:1–8. 10.1016/j.atherosclerosis.2009.12.027 20117784 PMC3924774

[122] Wang F Wang Y Kim MS Puthanveetil P Ghosh S Luciani DS. Glucose-induced endothelial heparanase secretion requires cortical and stress actin reorganization. Cardiovasc Res. (2010) 87:127–36. 10.1093/cvr/cvq051 20164120

[123] Chiu AP Wan A Lal N Zhang D Wang F Vlodavsky I. Cardiomyocyte VEGF regulates endothelial cell GPIHBP1 to relocate lipoprotein lipase to the coronary lumen during diabetes mellitus. Arterioscler Thromb Vasc Biol. (2016) 36:145–55. 10.1161/ATVBAHA.115.306774 26586663

[124] Zhang X Wang X Zhu H Kranias EG Tang Y Peng T. Hsp20 functions as a novel cardiokine in promoting angiogenesis via activation of VEGFR2. PLoS ONE. (2012) 7:e32765. 10.1371/journal.pone.0032765 22427880 PMC3299679

[125] Wang X Huang W Liu G Cai W Millard RW Wang Y. Cardiomyocytes mediate anti-angiogenesis in type 2 diabetic rats through the exosomal transfer of miR-320 into endothelial cells. J Mol Cell Cardiol. (2014) 74:139–50. 10.1016/j.yjmcc.2014.05.001 24825548 PMC4120246

[126] Biernacka A Frangogiannis NG. Aging and Cardiac Fibrosis. Aging Dis. (2011) 2:158–73. 21837283 PMC3153299

[127] Russo I Frangogiannis NG. Diabetes-associated cardiac fibrosis: cellular effectors, molecular mechanisms and therapeutic opportunities. J Mol Cell Cardiol. (2016) 90:84–93. 10.1016/j.yjmcc.2015.12.011 26705059 PMC4718740

[128] Cavalera M Wang J Frangogiannis NG. Obesity, metabolic dysfunction, and cardiac fibrosis: pathophysiological pathways, molecular mechanisms, therapeutic opportunities. Transl Res. (2014) 164:323–35. 10.1016/j.trsl.2014.05.001 24880146 PMC4180761

[129] Feng T Meng J Kou S Jiang Z Huang X Lu Z. CCN1-induced cellular senescence promotes heart regeneration. Circulation. (2019) 139:2495–8. 10.1161/CIRCULATIONAHA.119.039530 31107624

[130] Fujiu K Nagai R. Contributions of cardiomyocyte-cardiac fibroblast-immune cell interactions in heart failure development. Basic Res Cardiol. (2013) 108:357. 10.1007/s00395-013-0357-x 23740215

[131] Major JL McKinsey TA. Putting the heat on cardiac fibrosis, Hsp20 regulates myocyte-to-fibroblast crosstalk. JACC Basic Transl Sci. (2019) 4:200–3. 10.1016/j.jacbts.2019.03.007 31061922 PMC6488747

[132] Yuan J Liu H Gao W Zhang L Ye Y Yuan L. MicroRNA-378 suppresses myocardial fibrosis through a paracrine mechanism at the early stage of cardiac hypertrophy following mechanical stress. Theranostics. (2018) 8:2565–82. 10.7150/thno.22878 29721099 PMC5928909

[133] Accornero F Berlo JHV Benard MJ Lorenz JN Carmeliet P Molkentin JD. Placental growth factor regulates cardiac adaptation and hypertrophy through a paracrine mechanism. Circ Res. (2011) 109:272–80. 10.1161/CIRCRESAHA.111.240820 21636802 PMC3146170

[134] Frangogiannis NG. The functional pluralism of fibroblasts in the infarcted myocardium. Circ Res. (2016) 119:1049–51. 10.1161/CIRCRESAHA.116.309926 27789580 PMC5123771

[135] Datta R Bansal T Rana S Datta K Datta Chaudhuri R Chawla-Sarkar M. Myocyte-Derived Hsp90 modulates collagen upregulation via biphasic activation of STAT-3 in fibroblasts during cardiac hypertrophy. Mol Cell Biol. (2017) 37:e00611–16. 10.1128/MCB.00611-16 28031326 PMC5335508

[136] Chaturvedi P Kalani A Medina I Familtseva A Tyagi SC. Cardiosome mediated regulation of MMP9 in diabetic heart: role of mir29b and mir455 in exercise. J Cell Mol Med. (2015) 19:2153–61. 10.1111/jcmm.12589 25824442 PMC4568920

[137] Wang X Morelli MB Matarese A Sardu C Santulli G. Cardiomyocyte-derived exosomal microRNA-92a mediates post-ischemic myofibroblast activation both *in vitro* and *ex vivo*. ESC Heart Fail. (2020) 10.1002/ehf2.12584 31981320 PMC7083461

[138] Zhou S Tang X Chen HZ Sirtuins and insulin resistance. Front Endocrinol. (2018) 9:748 10.3389/fendo.2018.00748 PMC6291425 30574122

[139] Martin B Gabris-Weber BA Reddy R Romero G Chattopadhyay A Salama G. Relaxin reverses inflammatory and immune signals in aged hearts. PLoS ONE. (2018) 13:e0190935. 10.1371/journal.pone.0190935 29346407 PMC5773192

[140] Walaszczyk A Dookun E Redgrave R Tual-Chalot S Victorelli S Spyridopoulos I. Pharmacological clearance of senescent cells improves survival and recovery in aged mice following acute myocardial infarction. Aging Cell. (2019) 18:e12945. 10.1111/acel.12945 30920115 PMC6516151

[141] Roos CM Zhang B Palmer AK Ogrodnik MB Pirtskhalava T Thalji NM Chronic senolytic treatment alleviates established vasomotor dysfunction in aged or atherosclerotic mice. Aging Cell. (2016) 15:973–7. 10.1111/acel.12458 26864908 PMC5013022

